# Supplementary material for: Transmission network reconstruction for foot-and-mouth disease outbreaks incorporating farm-level covariates
Source: PLoS One. 2020 Jul 15;15(7):e0235660. doi: 10.1371/journal.pone.0235660 (PMC7363093; doi:10.1371/journal.pone.0235660)
Supplement: S1 File — (PDF) [file pone.0235660.s001.pdf]

Transmission network reconstruction for foot-and-mouth disease outbreaks incorporating farm-level covariates (Supplementary Materials)

Simon M. Firestone[1]*, Yoko Hayama[2], Max S. Y. Lau[3], Takehisa Yamamoto[2], Tatsuya Nishi[4], Richard A. Bradhurst[5], Haydar Demirhan[6], Mark A. Stevenson[1], Toshiyuki Tsutsui[2]

[1] Melbourne Veterinary School, Faculty of Veterinary and Agricultural Sciences, The University of Melbourne, Parkville, VIC 3010, Australia

[2] Viral Disease and Epidemiology Research Division, National Institute of Animal Health, National Agriculture Research Organization, Tsukuba, Ibaraki 305-0856, Japan

[3] Department of Biostatistics and Bioinformatics, Rollins School of Public Health, Emory University, Atlanta, Georgia, United States of America

[4] Exotic Disease Research Station, National Institute of Animal Health, National Agriculture and Food Research Organization, Kodaira, Tokyo, 187-0022, Japan

[5] Centre of Excellence for Biosecurity Risk Assessment, The University of Melbourne, Parkville, VIC 3010, Australia

[6] Mathematical Sciences Discipline, School of Science, RMIT University, Melbourne, VIC 3000, Australia

* Corresponding author: simon.firestone@unimelb.edu.au

S1 Simulated outbreak datasets: data and parameterisation

All simulated datasets are available here: https://doi.org/10.26188/5cf5e3af414a8

In accordance with data-sharing agreements for AADIS and the Miyazaki 2010 FMD outbreak dataset, random noise has been added to the coordinates of the farms in the datasets made publicly available.

Sellke threshold FMD outbreak parameterisations in Japan (J1–J50)

	J1-10	J11-20	J21-30	J31-40	J41-50	Reference/comment
n	50	100	150	200	400	Assumed outbreak size
n infected, median (range)	43 (36, 47)	88 (54, 93)	134 (92, 141)	139 (94, 169)	291 (132, 310)	Testing variety of scenarios
nt	7667	7667	7667	7667	7667	Genome length (nucleotides)
t_max	100	100	100	100	100	Maximum length of outbreak (days)
$\alpha \times 10^{-5}$	2.00	4.00	2.00	4.00	0.50	Primary transmission rate. Adjusted to scale background transmission
β	0.1	0.085	0.002	0.09	0.0015	Secondary transmission rate. Adjusted to scale β_{ij} and n(infected)
$\mu_1 \times 10^{-5}$	1.5	2.00	2.00	1.50	3.00	Rate of transitions. Cottam et al (2008)
$\mu_2 \times 10^{-5}$	0.10	0.20	0.10	0.10	0.50	Rate of transversions÷2. Cottam et al (2008) & Juleff et al (2013)
a	8	8	8	8	8	Shape parameter for Gamma distribution representing the latent period. Alexandersen et al (2003) & Haydon et al (2003)
b	0.5	0.5	0.5	0.5	0.5	Scale parameter for Gamma distribution representing the latent period.
c	12	15	15	12	21	Shape parameter for Exponential distribution representing the farm-level infectious period. Little prior information
κ	1.2	1.7	1.0	1.2	2	Spatial kernel scaling parameter. Bouma et al (2003)
p	0.1	0.1	0.2	0.1	0.2	Probability a base is mutated between consensus and common ancestor. Little prior information
ϕ_{pigs}	20	20	4	4	10	Infectivity of pig farms vs. cattle farms Alexandersen et al (2003)
ϕ_{other}	3	3	3	3	1	Infectivity of other farms vs. cattle farms
ρ_{pigs}	0.1	0.1	0.1	0.1	0.5	Susceptibility of pig farms vs. cattle farms
ρ_{other}	0.5	0.5	0.5	0.5	0.1	Susceptibility of other farms vs. cattle farms
ν	0.1	0.1	0.8	0.1	0.8	Effect of farm size on infectivity
τ	0.03	0.03	0.01	0.03	0.01	Effect of farm size on susceptibility
% cattle farms	60	64	38	50	54	
% pig farms	24	9	22	13	32	
% other farms	16	27	40	37	14	
Herd size, median (range)	285 (31, 2473)	276 (7, 7174)	289 (10, 8735)	256 (6, 39520)	252 (5, 13115)	

AADIS simulated FMD outbreaks in Australia (runs A1–A50)

In brief, they comprised the outputs of 50 runs of the Australian Animal Disease Spread (AADIS) hybrid model in its baseline configuration (Bradhurst et al., 2015), with movement restrictions and a stamping out only policy (i.e., no vaccination), each seeded on a large pig farm in central Victoria, Australia. Molecular sequence evolution was forwards simulated from a most recent common ancestor at the seed premises, designated with the 7667 nucleotide whole genome consensus sequence (O/JPN/2010-6/1S) sampled from the first farm presumed to be infected in the 2010 outbreak of foot-and-mouth disease in Miyazaki Prefecture of Japan (Nishi et al., 2017). Phylogenies were simulated with VirusTreeSimulator and SeqGen version 1.3.3 (Rambaut and Grass, 1997), parameterised based on empirical observations from the 2001 outbreak of FMD in the UK (Cottam et al., 2006, Cottam et al., 2008) and 2010 outbreak in Japan (Nishi et al., 2017), with a mutation rate of 2.168×10^{-5} mutations/site/day and a transition to transversion ratio of 7.61, with empirical nucleotide frequencies for the HKY model (Hasegawa et al., 1985) specified based on the observed data as (0.253, 0.282, 0.257, 0.209) for A, C, G and T, respectively.

References

Bradhurst, R. A., S. E. Roche, I. J. East, P. Kwan and M. G. Garner, 2015: A hybrid modeling approach to simulating foot-and-mouth disease outbreaks in Australian livestock. *Frontiers in Environmental Science,* 3, 17.

Cottam, E. M., D. T. Haydon, D. J. Paton, J. Gloster, J. W. Wilesmith, N. P. Ferris, G. H. Hutchings and D. P. King, 2006: Molecular epidemiology of the foot-and-mouth disease virus outbreak in the United Kingdom in 2001. *J. Virol.,* 80, 11274-11282.

Cottam, E. M., G. Thébaud, J. Wadsworth, J. Gloster, L. Mansley, D. J. Paton, D. P. King and D. T. Haydon, 2008: Integrating genetic and epidemiological data to determine transmission pathways of foot-and-mouth disease virus. *Proc. R. Soc. Lond. B Biol. Sci.,* 275, 887-895.

Hasegawa, M., H. Kishino and T.-a. Yano, 1985: Dating of the human-ape splitting by a molecular clock of mitochondrial DNA. *J Mol Evol,* 22, 160-174.

Nishi, T., M. Yamada, K. Fukai, N. Shimada, K. Morioka, K. Yoshida, K. Sakamoto, T. Kanno and M. Yamakawa, 2017: Genome variability of foot-and-mouth disease virus during the short period of the 2010 epidemic in Japan. *Vet Microbiol,* 199, 62-67.

Rambaut, A. and N. C. Grass, 1997: Seq-Gen: an application for the Monte Carlo simulation of DNA sequence evolution along phylogenetic trees. *Bioinformatics,* 13, 235-238.
