# Supplementary material for: Transmission network reconstruction for foot-and-mouth disease outbreaks incorporating farm-level covariates
Source: PLoS One. 2020 Jul 15;15(7):e0235660. doi: 10.1371/journal.pone.0235660 (PMC7363093; doi:10.1371/journal.pone.0235660)

Lau model (original) inferred transmission network in arbitrary space, differences from modified network in red.

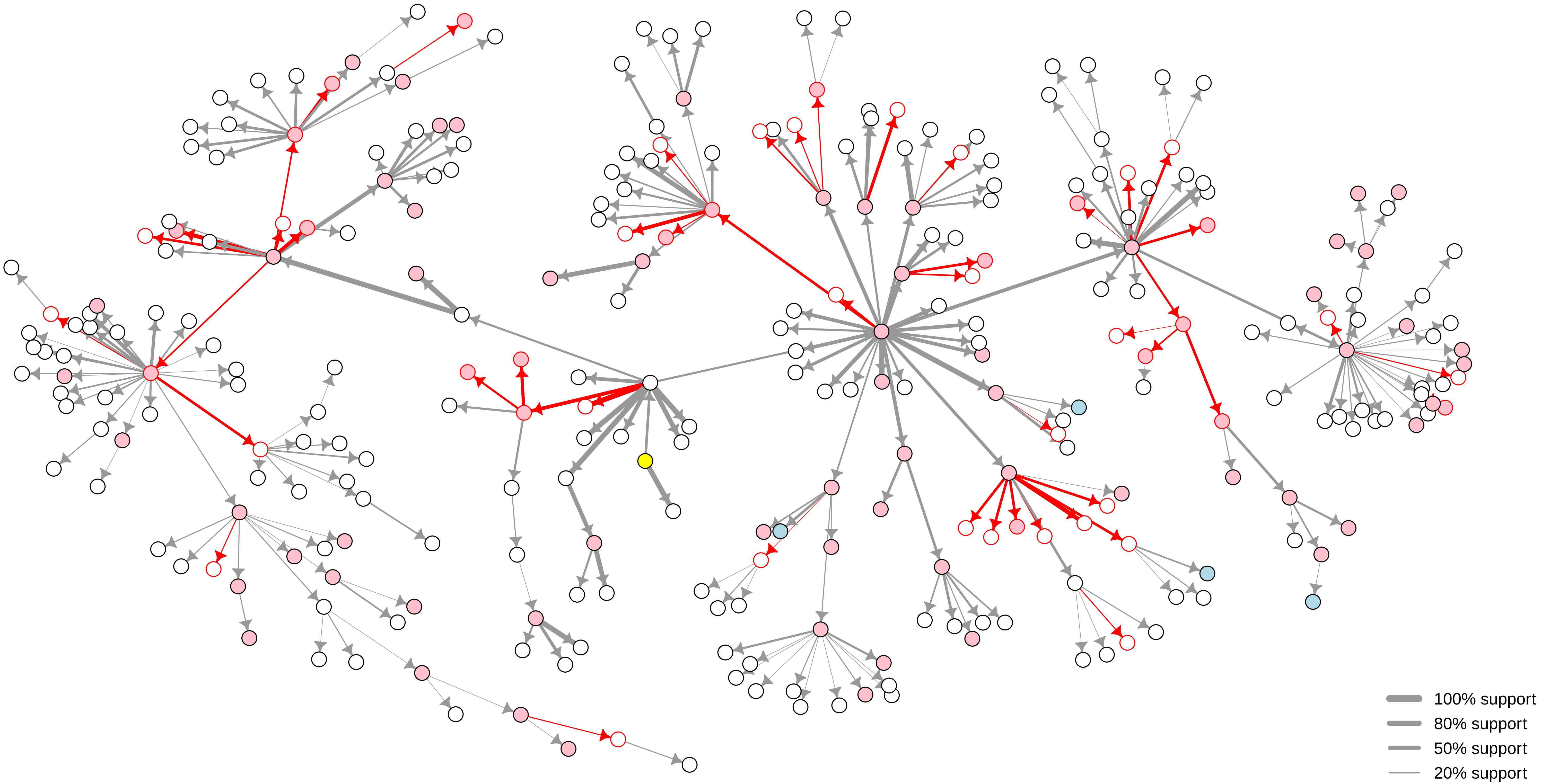

Supplement: S2 Fig — (PDF) [file pone.0235660.s003.pdf]
