# Supplementary material for: Transmission network reconstruction for foot-and-mouth disease outbreaks incorporating farm-level covariates
Source: PLoS One. 2020 Jul 15;15(7):e0235660. doi: 10.1371/journal.pone.0235660 (PMC7363093; doi:10.1371/journal.pone.0235660)
Supplement: S1 Table — (PDF) [file pone.0235660.s004.pdf]

Supplementary Materials, S2

Table S2: Comparison of the accuracy of inferences of Lau's joint Bayesian inference of the transmission network, for 100 simulated outbreaks of foot-and-mouth disease in Japan and Australia, detailed by run and model formulation.

Scenario[a]	Runs	n	Model[b]	Accuracy %, median (range) per scenario[c]			% of individuals with level of support, median (range)	
				Overall	>50% support	>80% support	>50% support	>80% support
Verification 1	J1-J10	50	original	68 (51, 86)	82 (56, 97)	97 (77, 100)	57 (42, 72)	34 (26, 58)
			modified	78 (66, 92)	86 (76, 97)	96 (88, 100)	68 (50, 84)	45 (30, 70)
Verification 2	J11-J20	100	original	72 (52, 81)	89 (71, 96)	98 (90, 100)	59 (44, 70)	42 (30, 56)
			modified	83 (64, 96)	92 (77, 98)	98 (93, 100)	72 (50, 80)	50 (39, 62)
Verification 3	J21-J30	150	original	69 (5, 78)	83 (5, 92)	95 (8, 99)	67 (50, 84)	47 (27, 54)
			modified	74 (10, 88)	86 (11, 93)	95 (13, 98)	76 (49, 87)	48 (31, 65)
Verification 4	J31-J40	200	original	70 (7, 83)	84 (8, 93)	95 (7, 98)	46 (33, 57)	30 (24, 36)
			modified	71 (12, 80)	86 (13, 96)	95 (12, 98)	47 (33, 63)	29 (20, 36)
Verification 5	J41-J50	400	original	77 (71, 83)	91 (86, 95)	96 (95, 98)	59 (50, 70)	43 (38, 48)
			modified	85 (79, 89)	93 (91, 96)	98 (95, 100)	62 (50, 72)	51 (38, 56)
Validation	A1- A50	12-98	original	74 (41, 92)	83 (53, 100)	93 (60, 100)	78 (46, 95)	56 (28, 72)
			modified	78 (31, 92)	85 (40, 100)	92 (83, 100)	83 (46, 100)	59 (31, 83)

[a] Runs J1-J50 were FMD outbreaks in Miyazaki Prefecture of Japan simulated in the same framework as the modified model, runs A1-A50 were FMD outbreaks in south-eastern Australia simulated in using the Australian Animal Disease Spread (AADIS) model (Bradhurst et al., 2015). [b] The original model is as described in (Lau et al., 2015, Firestone et al., 2019). The modified model incorporated additional terms for farm level transmissibility and susceptibility based on farm type and number of animals. [c] Accuracy was defined as the proportion of infected premises (IPs) for which the model-predicted most likely source (highest likelihood or most posterior support) was the true source. The denominator for accuracy at >50% and >80% support includes only those IPs for which the model-predicted most likely source attained that level of likelihood or posterior support.

References

Bradhurst, R. A., S. E. Roche, I. J. East, P. Kwan and M. G. Garner, 2015: A hybrid modeling approach to simulating foot-and-mouth disease outbreaks in Australian livestock. *Frontiers in Environmental Science,* 3, 17.

Firestone, S. M., Y. Hayama, R. Bradhurst, T. Yamamoto, T. Tsutsui and M. A. Stevenson, 2019: Reconstructing foot-and-mouth disease outbreaks: a methods comparison of transmission network models. *Sci. Rep.,* 9, 4809.

Lau, M. S., G. Marion, G. Streftaris and G. Gibson, 2015: A systematic Bayesian integration of epidemiological and genetic data. *PLoS Comput. Biol.,* 11, e1004633.
