# Supplementary material for: Transmission network reconstruction for foot-and-mouth disease outbreaks incorporating farm-level covariates
Source: PLoS One. 2020 Jul 15;15(7):e0235660. doi: 10.1371/journal.pone.0235660 (PMC7363093; doi:10.1371/journal.pone.0235660)
Supplement: S2 Table — (PDF) [file pone.0235660.s005.pdf]

S4 Nucleotide substitution model fit, FMD JPN 2010

Model	Parameters	BIC	AICc	lnL	I	G	TsTv	Freq A	Freq T	Freq C	Freq G
TN93+G	211	28855.92837	26413.74145	-12995.81381	n/a	0.130200216	9.076621971	0.25267511	0.207517529	0.282105132	0.25770223
TN93+G+I	212	28864.53158	26410.77085	-12993.32797	0.658211802	1.128512764	9.078792105	0.25267511	0.207517529	0.282105132	0.25770223
TN93+I	211	28874.867	26432.68008	-13005.28312	0.48313269	n/a	9.054655315	0.25267511	0.207517529	0.282105132	0.25770223
HKY+G	210	28884.22187	26453.60876	-13016.748	n/a	0.091456744	9.078205262	0.25267511	0.207517529	0.282105132	0.25770223
TN93	210	28886.71381	26456.1007	-13017.99397	n/a	n/a	9.039801569	0.25267511	0.207517529	0.282105132	0.25770223
HKY+G+I	211	28891.65401	26449.46709	-13013.67663	0.6945073	1.117198154	9.088293207	0.25267511	0.207517529	0.282105132	0.25770223
T92+G	208	28891.89381	26484.42833	-13034.15885	n/a	0.098889927	9.05254026	0.230096319	0.230096319	0.269903681	0.269903681
GTR+G	214	28895.57441	26418.66609	-12995.2745	n/a	0.130759886	7.514631099	0.25267511	0.207517529	0.282105132	0.25770223
T92+G+I	209	28899.50321	26480.46392	-13031.17612	0.688643473	1.117986426	9.056165217	0.230096319	0.230096319	0.269903681	0.269903681
GTR+G+I	215	28904.0168	26415.53469	-12992.70825	0.65793394	1.129782672	7.538650152	0.25267511	0.207517529	0.282105132	0.25770223
HKY+I	210	28906.01982	26475.40671	-13027.64698	0.48313269	n/a	9.049551648	0.25267511	0.207517529	0.282105132	0.25770223
GTR+I	214	28911.50824	26434.59991	-13003.24141	0.48313269	n/a	8.178632638	0.25267511	0.207517529	0.282105132	0.25770223
T92+I	208	28913.13531	26505.66983	-13044.7796	0.48313269	n/a	9.042822042	0.230096319	0.230096319	0.269903681	0.269903681
HKY	209	28918.7708	26499.73151	-13040.80991	n/a	n/a	9.03912873	0.25267511	0.207517529	0.282105132	0.25770223
T92	207	28925.68408	26529.79243	-13057.84143	n/a	n/a	9.03906144	0.230096319	0.230096319	0.269903681	0.269903681
GTR	213	28926.36894	26461.03442	-13017.45921	n/a	n/a	7.460939873	0.25267511	0.207517529	0.282105132	0.25770223
K2+G	207	28926.5078	26530.61616	-13058.25329	n/a	0.096171882	9.054583311	0.25	0.25	0.25	0.25
K2+G+I	208	28934.03521	26526.56974	-13055.22956	0.690861506	1.115134899	9.059015367	0.25	0.25	0.25	0.25
K2+I	207	28947.95835	26552.06671	-13068.97857	0.48313269	n/a	9.043104122	0.25	0.25	0.25	0.25
K2	206	28960.5753	26576.25749	-13082.07449	n/a	n/a	9.039041382	0.25	0.25	0.25	0.25
JC+G	206	29309.27585	26924.95803	-13256.42476	n/a	0.096748703	0.5	0.25	0.25	0.25	0.25
JC+G+I	207	29316.84345	26920.9518	-13253.42112	0.690282044	1.116562183	0.5	0.25	0.25	0.25	0.25
JC+I	206	29330.67429	26946.35648	-13267.12398	0.48313269	n/a	0.5	0.25	0.25	0.25	0.25
JC	205	29343.27176	26970.52778	-13280.21016	n/a	n/a	0.5	0.25	0.25	0.25	0.25

All positions containing gaps and missing data were eliminated. There were a total of 7559 positions in the final dataset. Evolutionary analyses were conducted in MEGA7[1,2].

Abbreviations: Bayesian Information Criterion (BIC); Akaike Information Criterion corrected (AICc); Maximum negative log Likelihood value (lnL); Proportion invariant (I); Discretised Gamma distribution shape parameter (G); Transition to transversion ratio (TsTv); Empirically estimated frequencies

References

1. Nei M. and Kumar S. (2000). Molecular Evolution and Phylogenetics. Oxford University Press, New York.
2. Kumar S., Stecher G., and Tamura K. (2016). MEGA7: Molecular Evolutionary Genetics Analysis version 7.0 for bigger datasets.Molecular Biology and Evolution 33:1870-1874.
