# Supplementary material for: Transmission network reconstruction for foot-and-mouth disease outbreaks incorporating farm-level covariates
Source: PLoS One. 2020 Jul 15;15(7):e0235660. doi: 10.1371/journal.pone.0235660 (PMC7363093; doi:10.1371/journal.pone.0235660)
Supplement: S3 Table — (PDF) [file pone.0235660.s006.pdf]

S6: SCOTTI model source support

IP	P(source sampled)	Normalised support for proposed source
1	0.334	0.428
2	0.258	0.481
3	0.344	0.491
5	0.107	0.689
6	0.024	0.469
7	0.249	0.732
8	0.235	0.475
9	0.601	0.795
10	0.368	0.546
11	0.381	0.544
12	0.209	0.263
13	0.230	0.278
14	0.039	0.236
15	0.248	0.231
16	0.143	0.262
19	0.322	0.190
20	0.532	0.309
22	0.240	0.166
31	0.315	0.267
36	0.357	0.158
37	0.703	0.783
40	0.320	0.356
44	0.331	0.181
48	0.318	0.343
50	0.236	0.213
53	0.230	0.237
57	0.128	0.218
65	0.178	0.185
66	0.340	0.318
67	0.220	0.192
68	0.601	0.911
69	0.223	0.178
70	0.134	0.194
71	0.156	0.176
72	0.152	0.220
74	0.351	0.357
75	0.345	0.144
76	0.367	0.190
79	0.538	0.891
81	0.325	0.125
82	0.198	0.194
83	0.207	0.171
84	0.332	0.169
85	0.137	0.195
86	0.383	0.503
87	0.254	0.142
88	0.236	0.170
89	0.253	0.145
90	0.349	0.131
91	0.241	0.175
92	0.218	0.196
95	0.997	0.146
96	0.216	0.177
97	0.319	0.143
98	0.254	0.342
99	0.338	0.737
100	0.114	0.191
101	0.290	0.139
102	0.226	0.167
103	0.204	0.172
104	0.347	0.129
107	0.317	0.146
109	0.277	0.137
110	0.998	0.285
111	0.998	0.175
112	0.077	0.278
113	0.289	0.223
115	0.998	0.194
116	0.296	0.494
117	0.202	0.175
118	0.338	0.303
119	0.196	0.159
121	0.190	0.176
124	0.212	0.168
125	0.998	0.189
129	0.998	0.210
131	0.998	0.162
132	0.998	0.159
136	0.198	0.170
139	0.999	0.214
140	0.140	0.182
145	0.251	0.146
147	0.410	0.318
149	0.251	0.149
150	0.996	0.168
151	0.998	0.294
158	0.996	0.218
161	0.294	0.224
163	0.998	0.781
164	0.999	0.182
165	0.999	0.248
166	0.999	0.482
167	0.132	0.201
168	0.999	0.197
169	0.999	0.198
182	0.997	0.175
204	0.999	0.260
206	0.999	0.381
211	0.997	0.330
235	0.999	0.350
265	0.999	0.281
280	0.999	0.340
283	0.999	0.251
288	0.999	0.413
